# Three-dimensional finite element analysis of extra short implants focusing on implant designs and materials

**DOI:** 10.1186/s40729-019-0202-6

**Published:** 2020-01-29

**Authors:** Haruka Araki, Tamaki Nakano, Shinji Ono, Hirofumi Yatani

**Affiliations:** Department of Fixed Prosthodontics, Osaka University Graduate School of Dentistry, Osaka, 565-0871 Japan

**Keywords:** Short dental implant, Titanium–zirconium alloy, Tissue level implant, Finite element analysis, Biomechanics

## Abstract

**Aim:**

When using short implants, fracture of the implant body and bone resorption are a concern because stress concentrates on and around a short implant. The purpose of this research is to investigate the differences in stress distribution between tissue level (TL) and bone level (BL) implant body designs, and between commercially pure titanium (cpTi) and the newer titanium–zirconium (TiZr) alloy in using short implants.

**Materials and methods:**

Models of TL and BL implants were prepared for three-dimensional finite element analysis. The implants were produced in 10 mm, 8 mm, and 6 mm lengths, and the TL was also produced in a 4-mm length. A static load of 100 N inclined at 30° to the long axis was applied to the buccal side of the model. The largest maximum principal stress value in the cortical bone and the largest von Mises stress value in the implant body were evaluated.

**Results:**

Stress concentration was observed at the connection part of the implant, especially above the bone in TL and within the bone in BL. In the TL design, tensile stress occurred on the buccal side and compressive stress on the lingual side of the cortical bone. Conversely, in the BL design, tensile stress occurred on the lingual side of the cortical bone. CpTi and TiZr showed a similar stress distribution pattern. The maximum stress values were lower in the TL design than the BL design, and they were lower with TiZr than cpTi for both the cortical bone and implant body. The maximum value tended to increase as the length of the implant body decreased. In addition, the implant body design was more influential than its length, with the TL design showing a stress value similar to the longer BL design.

**Conclusion:**

Using TiZr and a TL design may be more useful mechanically than cpTi and a BL design when the length of the implant body must be shorter because of insufficient vertical bone mass in the mandible.

## Summary

Dental implants are widely used as a treatment option to replace a defective prosthesis. In recent years, treatment using short implants, which are ≤ 8 mm in length, has been increasing in cases with vertical bone resorption [1]. It is thought that this will become more popular as the number of patients who require minimally invasive treatment, such as older patients and those with chronic disease, is increasing [2–5].

There are two major implant designs; tissue level (TL) implants, where the platform is located under the soft tissue level, and bone level (BL) implants, where the platform is placed at the crestal bone level. TL implants are often avoided in the esthetic area, but there are no clear criteria for the selection of either implant design. Conversely, TL implants are more structurally favorable for a shorter implant body than BL implants because of the submerged design and shorter abutment screw. Clinically, the 4-mm-long TL implant is the shortest used [6, 7]. However, no report has been previously undertaken on the difference between the mechanical behavior of TL and BL implants, so it is not clear which design is more advantageous.

Recently, a titanium–zirconium alloy (TiZr) has been developed, which contains approximately 15% zirconium in titanium and has high biocompatibility, similar to commercially pure titanium (cpTi). Furthermore, TiZr has higher mechanical strength when compared with cpTi and is expected to be effective for preventing fracture of the implant body [8–11]. Clinically, it has been reported that TiZr implants have no significant difference in marginal bone resorption and survival rate when compared with cpTi implants, and their use is equivalent to cpTi implants [12–14]. However, the difference in stress distribution to the surrounding bone and within the implant body between cpTi and TiZr implants has not been elucidated.

Finite element analysis (FEA) is often used to predict the long-term prognosis of a device in an intraoral environment simulating loading conditions [15–17]. Therefore, we conducted a mechanical study of implant using three-dimensional FEA, with the purpose of clarifying the differences between cpTi and TiZr implants, TL and BL implants, and their length.

## Materials and methods

**Fig. 1:**
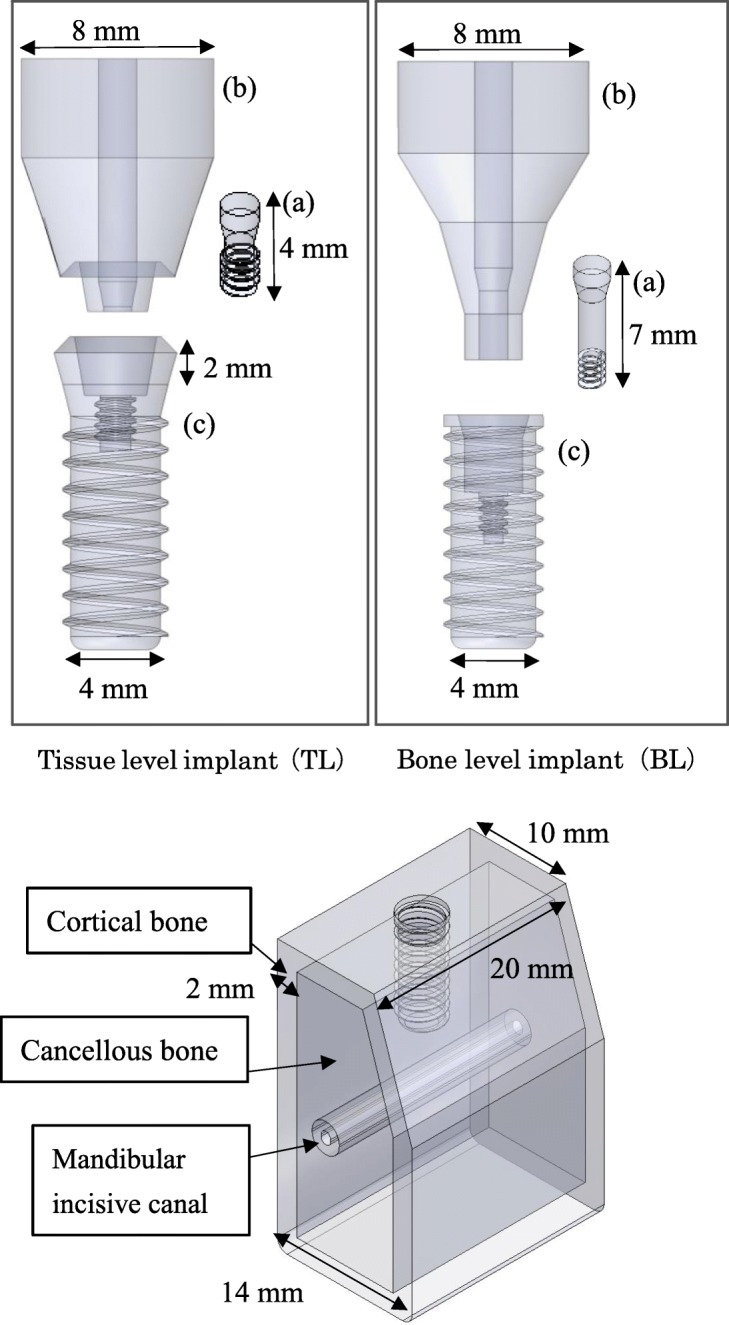
Three-dimensional CAD model. (upper: **a** abutment screw, **b** superstructure, **c** implant body; Lower: bone model)

**Fig. 2:**
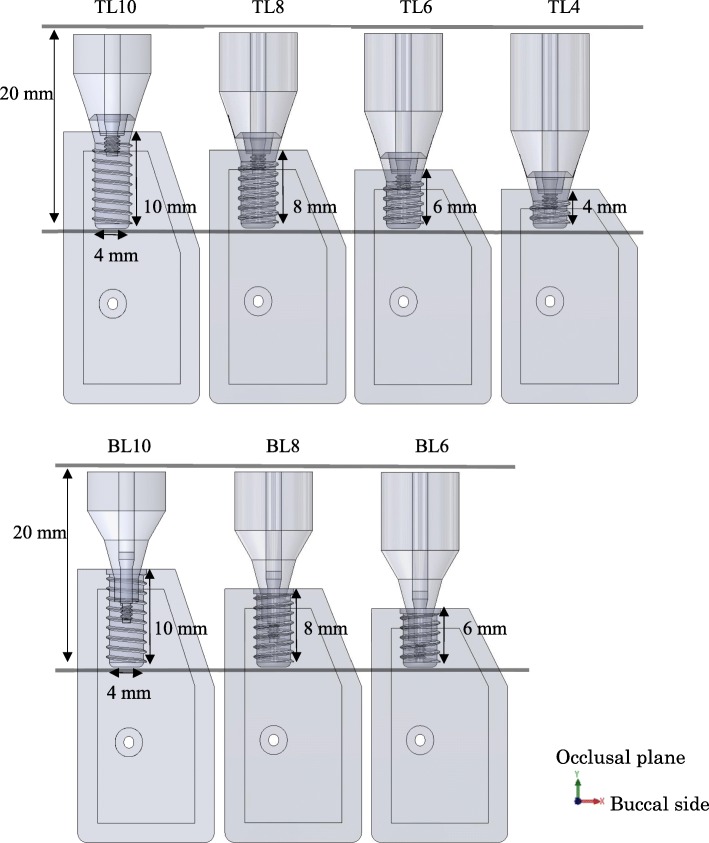
Models of different implant body lengths

**Fig. 3:**
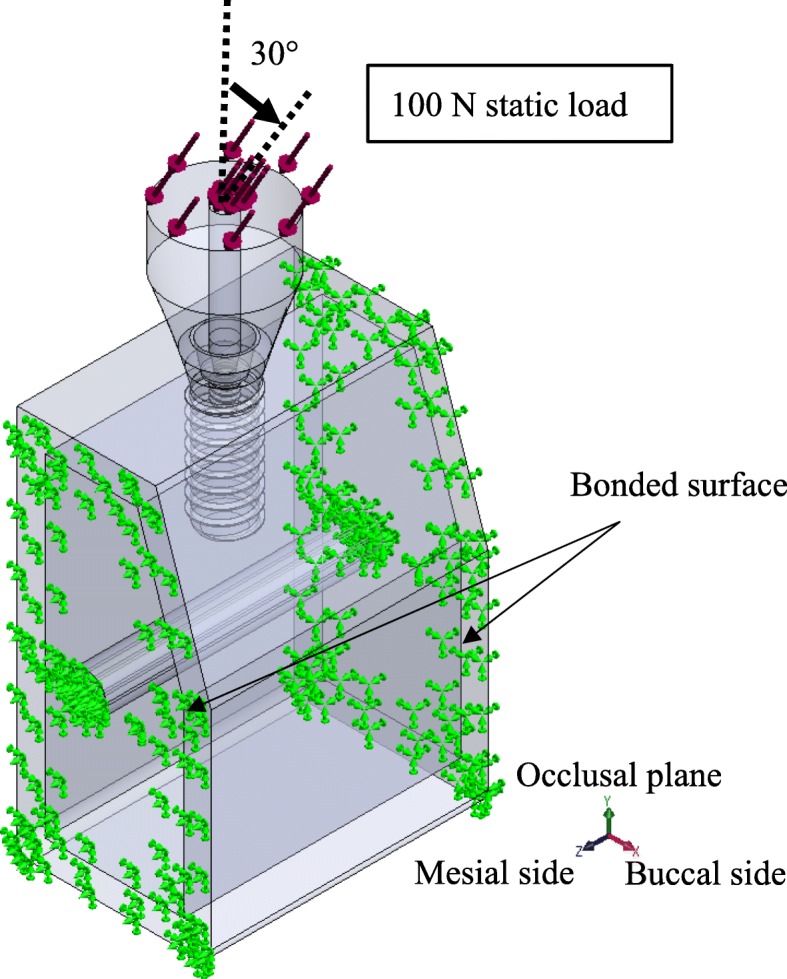
Assembly of implant and bone models. A static load of 100 N was applied obliquely from the buccal side to the occlusal plane of the superstructure at 30 to the long axis of the implant

TL and BL three-dimensional computer-aided design (CAD) implant models were created using the CAD function in computer-aided engineering software (SolidWorks 2014, Dassault Systèmes SolidWorks Corporation, MA, USA), and they were created with reference to conical connection implant used clinically. The connection part of superstructure has a tapered 15° conical shape without any special locking mechanism. The length of each part is as shown in Fig. 1. The diameter of the implant bodies was 4.1 mm. The lengths of the TL models were 10.0 mm, 8.0 mm, 6.0 mm, and 4.0 mm; the lengths of the BL models were 10.0 mm, 8.0 mm, and 6.0 mm (Fig. 2). A CAD model of mandibular molar alveolar bone with 2.0-mm-wide cortical bone was prepared, in which each implant CAD model was embedded. The implant and superstructure were connected with an abutment screw. The distance from the occlusal plane to the apex of the implant was 20.0 mm in any given model; that is, the crown–implant ratio increased with decreasing implant body length. To simulate osseointegration, a “fixed bond” condition was set at the implant body–bone interface. A “contact” condition with friction coefficient 0.3 was set at the interface between the implant components, which facilitated microscopic sliding. The mesial and distal sides of the mandibular alveolar bone were fixed. A static load of 100 N was applied obliquely from the buccal side to the occlusal plane of the superstructure at 30° to the long axis of the implant (Fig. 3) [18]. The mechanical properties of the components used in this research study are shown in Table 1, and the implant body used a value obtained by performing a compression test in advance. Tetrahedral elements were used for the FEA, and the number of elements was determined by conducting a convergence test based on the maximum principal stress. The distribution and the maximum von Mises stress value were measured in the implant body, and the distribution and largest maximum principal stress value were measured in the cortical bone.

**Table 1:** Mechanical properties of each model component

	Young’s modulus (GPa)	Poisson’s ratio	Reference
Abutment screw (Ti-6Al-4V)	110	0.33	[19]
Superstructure (gold alloy)	96.6	0.35	[20]
Cortical bone	13	0.3	[21]
Cancellous bone	1.37	0.3	[21]
Implant body (cpTi)	110	0.34	
Implant body (TiZr)	97.3	0.36	

To validate the accuracy of the FEA model, microstrain of the surrounding bone were compared with the results of in vitro experiment measured with strain gauge [22]. In the literature, it was reported that microstrain of 59.3876 ± 24.7185 μe at the neck of implant and 17.3456 ± 12.9147 μe at the apical occurred in a bovine bone under an oblique load of 120 N. Under the same condition as the literature, the microstrain of BL10 was 70.6 μe at the neck and 7.741 μe at the apical, which was within the standard deviation of the results of the literature. As a result, the present FEA study may be considered to have acceptable resemblance to past literature.

## Results

### Cortical bone stress

**Fig. 4:**
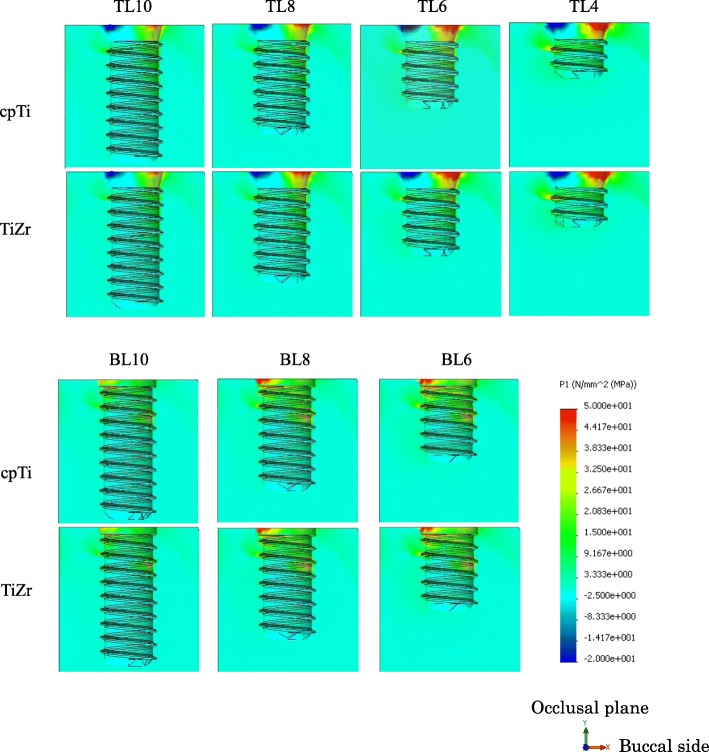
Distribution of the maximum principle stress in the surrounding bone (right: buccal side, left: lingual side)

**Fig. 5:**
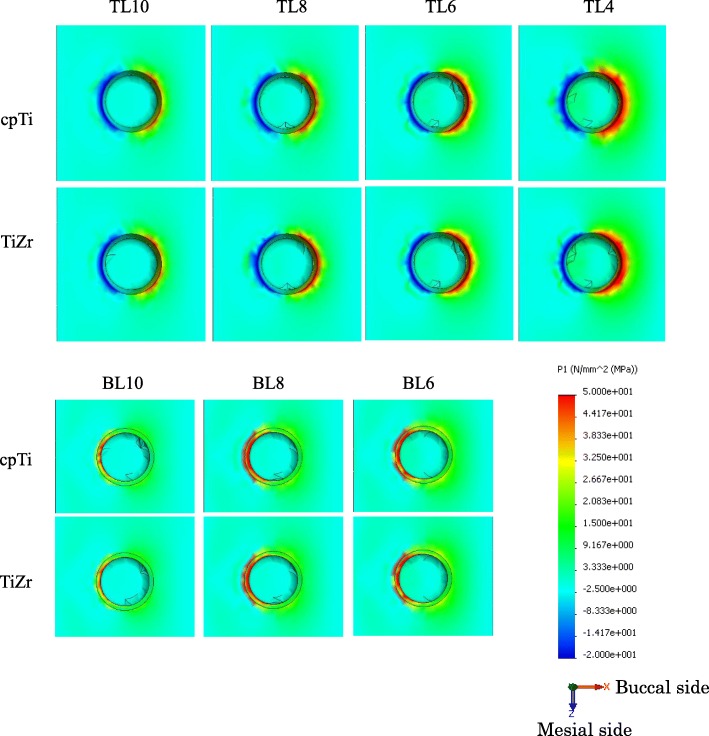
Distribution of the maximum principle stress in the surrounding bone (occlusal view)

**Fig. 6:**
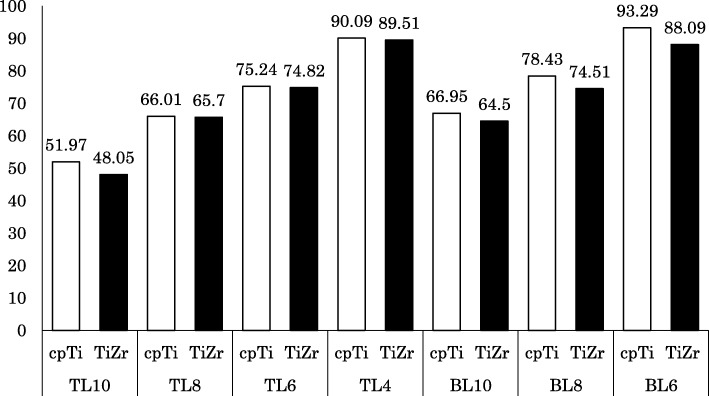
Largest maximum principle stress value in cortical bone (MPa)

The distribution of the maximum principal stress in the cortical bone concentrated on the neck of the implant body. In the TL implants, tensile stress was concentrated on the buccal side and compressive stress on the lingual side. In the BL implants, tensile stress concentration was observed on the lingual side. The distributions were similar between the cpTi and TiZr implants (Figs. 4 and 5). The maximum stress value of the TL implants was smaller than that of the BL implants, and TiZr revealed a smaller value than cpTi in the same length. Regardless of the implant body design and material, the stress concentration increased as the implant length decreased (Fig. 6). The TL implants revealed a comparable maximum stress value to 2.0 mm longer BL implants.

### Implant body stress

**Fig. 7:**
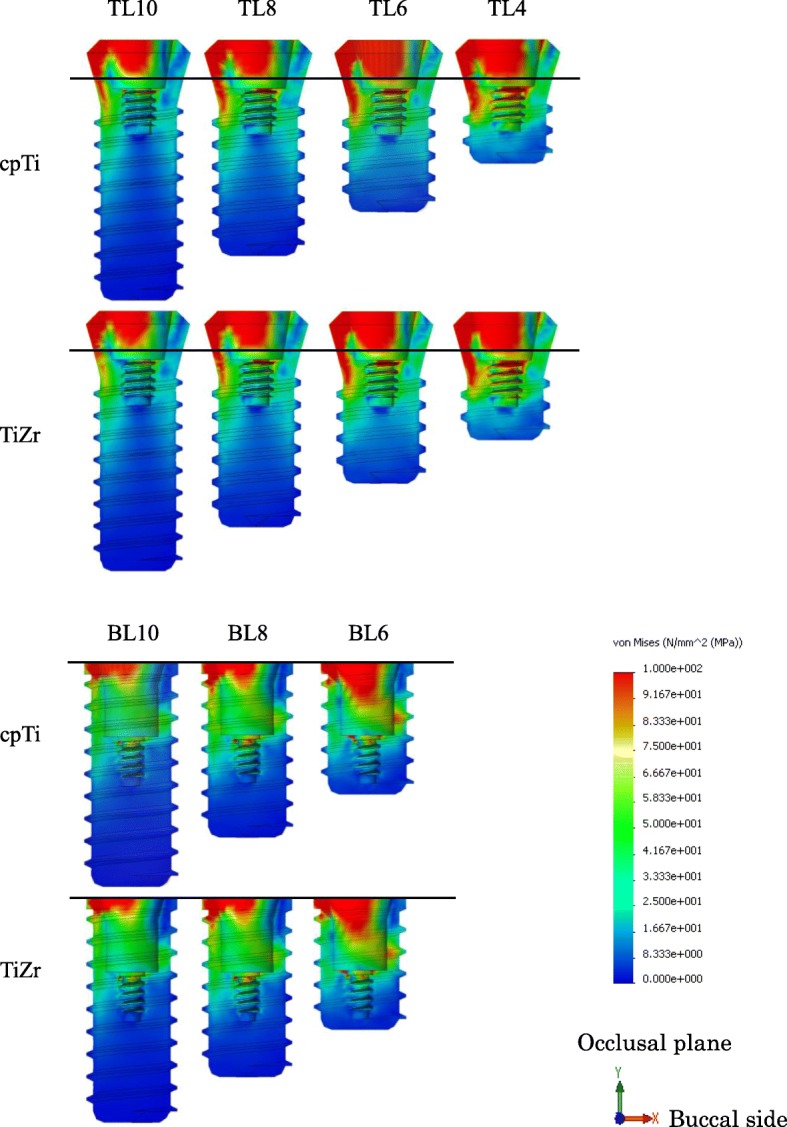
Von Mises stress distribution in implant bodies. (*right*: buccal side, *left*: lingual side)

**Fig. 8:**
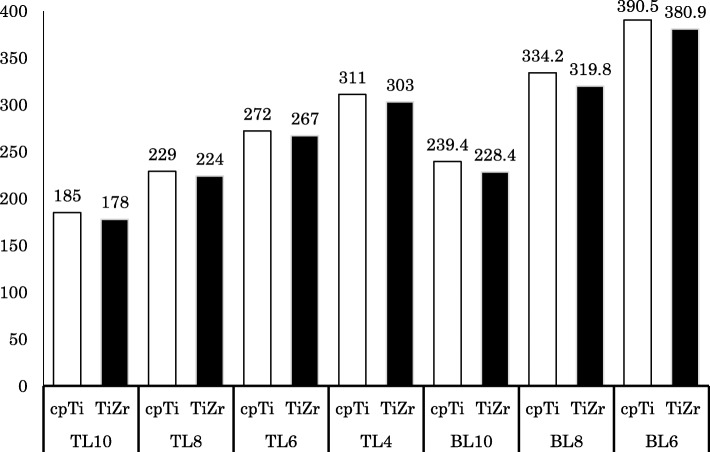
Maximum von Mises stress value in implant bodies (MPa)

The distribution of von Mises stress in the implant component was concentrated on the connection part of the implant, which was above the bone in the TL design and under the bone in the BL design. In the implant body, stress concentration was observed on the lingual side, and stress propagated along the interface with the superstructure (Fig. 7). The maximum von Mises stress value of the TL implants was smaller than that of the BL implants, and TiZr revealed a smaller value than cpTi in the same length. Regardless of the design or material used, the stress concentration increased with decreasing implant length (Fig. 8). The 4.0 mm TL implant revealed the same amount of stress as the 8.0 mm BL implant, suggesting considerably lower stress in the TL versus BL design.

## Discussion

Overloading, which is one of the factors contributing to bone resorption around an implant body, can lead to complications because force is applied beyond the prosthodontic or biological tolerance [23]. It is believed that when stress of a certain magnitude is applied to the bone, microscopic bone destruction occurs resulting in bone resorption [24, 25]. Because implants do not have buffering mechanisms like a periodontal ligament, occlusal force propagates directly to the surrounding bone via the implant body. Therefore, overloading is considered to be deeply involved in bone resorption after the beginning of functional loading [26]. In addition, since it has been reported that bone resorption by overloading is facilitated by infection, a design that does not place excessive stress on the surrounding bone is ideal [27]. Using a strain gauge is a popular method to evaluate stress in vivo. However, since the implant body is embedded in bone, it is impossible to evaluate the stress within the implant body noninvasively using the strain gauge method. Also, because the sensor is large, it may not be suitable for capturing the stress distribution to each part of the tissue. However, FEA is a method of capturing the entire behavior by dividing an object having a complicated shape or property into simple small parts and performing a numerical calculation. It is advantageous that an appropriate analysis model can represent the stress distribution within a structure noninvasively.

There are various types of stress that can be measured, and this must be selected according to the material and the item under evaluation. In this study, the implant body was either cpTi or TiZr, and this was evaluated using the von Mises stress, which is proportionally equivalent to the “shear strain energy theory”. Fracture occurs when the principal stress, which is the maximum generated stress, exceeds the strength of the material. Brittle materials are generally evaluated using the “maximum principal stress theory”; therefore, the maximum principal stress was used for evaluating the cortical bone in this study. It has been reported that the bone is strongly resistant to compressive stress, and the bone resorption threshold of tensile stress is lower than that of compressive stress by about 30% [28]. However, one study reported that tensile stress promotes bone deposition and compressive stress promotes bone resorption [29]. Therefore, the maximum absolute value of the maximum principal stress was measured, and the stress type (tensile or compressive) that had the greater maximum principal stress was then evaluated.

The difference in the implant body structure between the submerged and non-submerged implants greatly affected the stress distribution. Since the TL implant body lies above the bone level rather than level with the crestal bone, it was found that the stress concentrates above the apex of the alveolar bone, regardless of the material type. As a result, the maximum stress value in the cortical bone was found to be lower in the TL versus BL design, and it was suggested that TL implants may have a lower risk of bone resorption than BL implants. Regarding the difference in stress distribution in cortical bone, in the TL design, tensile stress was generated on the buccal side and compressive stress was generated on the lingual side because of the rotational moment of implant body caused by lateral loading. The BL design was influenced by the stress generated at the interface between the superstructure and the implant body in lingual side generated less rotational moment. Because the connection with the implant body is conical, the compressive stress generated on the lingual side of the superstructure by the rotational moment was transferred to internal stress within the implant body. It was believed that only the tensile component in the resulting compressive stress was transmitted to the implant body and then propagated to the cortical bone. In the buccal side, the large stress was not generated at the interface between the implant body and the superstructure, so stress concentration in cortical bone was not seen. In the lingual side, the tensile stress generated in the implant body was transmitted. Therefore, stress distribution in the cervical cortical bone was affected by the implant body design.

CpTi has high corrosion resistance and biocompatibility and is widely used as a biomaterial. However, its tensile and fatigue strengths are considered to be insufficient, and the development of a biomaterial with increased strength has been attempted [30, 31]. Among these materials, Ti-6Al-4V alloys containing 6% and 4% aluminum and vanadium, respectively, are widely used for dental implants. This material shows mechanical strength exceeding that of cpTi and is used in part for small diameter and short implants where large loads are expected. Aluminum is an element that can cause neurotoxicity and vanadium is cytotoxic; therefore, the biocompatibility of this alloy is inferior to cpTi [30–33]. Thus, it is difficult to combine both mechanical strength and the biocompatibility necessary for biomaterials that function for a long time in vivo. TiZr, which has been used in recent years, is not expected to be toxic and has high mechanical strength exceeding Ti-6Al-4V, so it is anticipated as a new biomaterial. Based on in vivo experiments, its osseointegration and biocompatibility are comparable to cpTi [34–36]. Other studies reported that TiZr has a tensile strength 40% higher and a fatigue strength 13–42% higher than cpTi, as well as increased mechanical strength when compared with conventional biomaterials [8]. In addition, a low elastic modulus is important as a mechanical property for implants to reduce stress at the implant body–bone interface [32]. On average, the elastic moduli of cpTi and cortical bone are 110 GPa and 10 GPa, respectively. When the elastic moduli differ greatly like this, the strain generated at the interface differs, so that high stress is generated at both interfaces. Therefore, by reducing the elastic modulus of the implant body, it is possible to reduce the amount of stress generated. In this study, TiZr was found to have a smaller maximum value than cpTi for both the cortical bone and implant body in both the TL and BL designs because the elastic modulus of TiZr is 10% smaller than that of cpTi, and its Poisson’s ratio is larger than cpTi. In the cortical bone, the lower elastic modulus of the implant body reduced the difference in that modulus between the implant body and bone, resulting in reduced stress generation at the implant body–bone interface. In the implant body, the Poisson’s ratio became larger in TiZr than in cpTi, and the strain of the implant body itself increased, resulting in decreased stress. This result is consistent with the report that stress in the surrounding bone decreases as the elastic modulus diminishes [36]. It has also been reported that as the elastic modulus decreases, surrounding bone formation increases. It has been found that TiZr is more favorable than cpTi with respect to stress distribution in the cortical bone and implant body when overloading occurs [31, 37].

Clinically, it is generally considered that the crown length increases proportionally when the length of the implant body decreases because of alveolar bone resorption. However, most previous studies performing FEA of short implants have analyzed them with a standard crown length [38]. In this study, the distance from the tip of the implant body to the occlusal plane was standardized to make the analysis condition more applicable to the clinical situation. We set the condition that the crown length would increase as the length of the implant body decreased, and the analysis was then performed. As the crown–implant ratio increases, the rotational moment increases and the stress generated at the implant neck also increases [39]. Previous research reported no significant difference in the survival rate and bone resorption when the crown–implant ratio ranges from 2:1 to 3:1, but when an implant of 4.0 mm length is inserted, it is assumed that the ratio increases [39, 40]. Another report suggested that clinical outcomes are significantly worsened if the crown length exceeds 15.0 mm. In consideration of these reports, analysis of the 4.0 mm length implant was carried out with a crown length of 16.0 mm and a crown–implant ratio of 4:1 [41].

Regarding the FEA of short implants, it is already known that stress concentrates on the cervical cortical bone regardless of the length of the implant body [42]. The present study found that the stress distribution in the cervical cortical bone increased as the length of the implant body decreased. In addition, BL implants showed a maximum stress value similar to TL implants that were 2 mm shorter than the BL design. As such, it was found that the difference in design between TL and BL implants has a greater influence on stress distribution than the 2-mm difference in length. Good clinical results have been reported for BL implants with a length of 6.0 mm. Furthermore, the appropriate crown length and crown–implant ratio have never been evaluated clinically for a 4.0-mm-long TL implant; however, in our in vitro experiment, this implant was placed under severe loading conditions and the maximum stress values were similar between the 6-mm-long BL implant and the 4-mm-long TL implant. Therefore, it is suggested that the 4-mm-long TL implant may be mechanically useful [43]. Although the cases to consider the use of short implants should be selected carefully, it was suggested that the risk of failure can be reduced by the design and material selection of the implant body. However, especially, in extra short implants, these stress concentration increases mechanical risks such as fracture of the implant body and screw loosening. The results in this study are under the limited conditions to compare the stress distribution in the surrounding bone. In the future, in addition to need to evaluate the stress distribution of the component, we believe that it will be necessary to compare with the accumulated clinical results.

## Conclusion

Within the limitations of this study, the following conclusions were drawn.

- The stress distribution in the cortical bone and implant body was smaller in the TL implant than in the BL implant.
- The TiZr alloy had a lower elastic modulus than cpTi, and the stress distribution generated in the cortical bone and implant body was also lower.
- The stress distribution generated in the cortical bone and the implant body increased as the length of the implant body decreased, but the design of the implant body had a greater influence than the implant body length.

## Data Availability

Not applicable
